# Influence of the microbiome, diet and genetics on inter-individual variation in the human plasma metabolome

**DOI:** 10.1038/s41591-022-02014-8

**Published:** 2022-10-10

**Authors:** Lianmin Chen, Daria V. Zhernakova, Alexander Kurilshikov, Sergio Andreu-Sánchez, Daoming Wang, Hannah E. Augustijn, Arnau Vich Vila, Lifelines Cohort Study, Rinse K. Weersma, Marnix H. Medema, Mihai G. Netea, Folkert Kuipers, Cisca Wijmenga, Alexandra Zhernakova, Jingyuan Fu

**Affiliations:** 1 Department of Genetics, University of Groningen, University Medical Center Groningen, Groningen, the Netherlands; 2 Department of Pediatrics, University of Groningen, University Medical Center Groningen, Groningen, the Netherlands; 3 Department of Cardiology, Nanjing Medical University, The First Affiliated Hospital of Nanjing Medical University, Nanjing, China; 4 Cardiovascular Research Center, The Affiliated Suzhou Hospital of Nanjing Medical University, Suzhou Municipal Hospital, Gusu School, Nanjing Medical University, Suzhou, China; 5 Laboratory of Genomic Diversity, Center for Computer Technologies, ITMO University, St. Petersburg, Russia; 6 Bioinformatics Group, Wageningen University, Wageningen, the Netherlands; 7 Department of Gastroenterology and Hepatology, University of Groningen, University Medical Center Groningen, Groningen, the Netherlands; 8 Department of Internal Medicine and Radboud Center for Infectious Diseases, Radboud University Medical Center, Nijmegen, the Netherlands; 9 Department of Immunology and Metabolism, Life and Medical Sciences Institute, University of Bonn, Bonn, Germany; 10 European Research Institute for the Biology of Ageing, University of Groningen, University Medical Center Groningen, Groningen, the Netherlands

**Keywords:** Genetics, Microbial communities, Metagenomics, Epidemiology

## Abstract

The levels of the thousands of metabolites in the human plasma metabolome are strongly influenced by an individual’s genetics and the composition of their diet and gut microbiome. Here, by assessing 1,183 plasma metabolites in 1,368 extensively phenotyped individuals from the Lifelines DEEP and Genome of the Netherlands cohorts, we quantified the proportion of inter-individual variation in the plasma metabolome explained by different factors, characterizing 610, 85 and 38 metabolites as dominantly associated with diet, the gut microbiome and genetics, respectively. Moreover, a diet quality score derived from metabolite levels was significantly associated with diet quality, as assessed by a detailed food frequency questionnaire. Through Mendelian randomization and mediation analyses, we revealed putative causal relationships between diet, the gut microbiome and metabolites. For example, Mendelian randomization analyses support a potential causal effect of *Eubacterium rectale* in decreasing plasma levels of hydrogen sulfite—a toxin that affects cardiovascular function. Lastly, based on analysis of the plasma metabolome of 311 individuals at two time points separated by 4 years, we observed a positive correlation between the stability of metabolite levels and the amount of variance in the levels of that metabolite that could be explained in our analysis. Altogether, characterization of factors that explain inter-individual variation in the plasma metabolome can help design approaches for modulating diet or the gut microbiome to shape a healthy metabolome.

## Abstract (web summary)

The influence of an individual’s genetics, diet and gut microbiome on their plasma metabolome was studied in 1,368 individuals and Mendelian randomization and mediation analyses were used to unveil causal relationships between diet, the gut microbiome and the metabolome.

## Main

The plasma metabolome represents a functional readout of metabolic activities within different organs and tissues of the body. Levels of specific plasma metabolites may therefore reflect the presence of specific diseases or an individual’s susceptibility to developing complex metabolic diseases such as cardiovascular and kidney disorders, diabetes, cancers and Crohn’s disease^1^. Elucidating the genetic, dietary and microbial factors that shape human metabolism is crucial for understanding the origin and determinants of plasma metabolites, and hence for the eventual design of intervention strategies aimed at a healthy metabolome.

Inter-individual variations in the human plasma metabolome have already been linked to genetics, diet and the gut microbiome in several cohort-based studies^1–4^. For instance, a reference map of potential determinants of the human serum metabolome was established in 491 individuals from an Israeli cohort, and the authors reported 335 metabolites that were significantly explained by diet and 182 that were explained by the gut microbiome^3^. More recently, the Personalized Responses to Dietary Composition Trial assessed the impact of diet and the microbiome on host metabolism in 1,098 individuals from the United Kingdom and United States and observed that the microbial species associated with healthy dietary habits overlapped with those associated with favorable cardiometabolic and postprandial markers^4^.

As diet, genetics and the gut microbiome are highly heterogeneous between different countries, we aimed to: (1) systematically identify dietary, genetic and microbial factors that are associated with plasma metabolites; (2) identify which of the three factors (diet, genetics or the microbiome) explains the most inter-individual variability in metabolites compared with the other two (the dominant factor); and (3) assess their causal relationships using in silico approaches. To do so, we quantified the plasma levels of 1,183 metabolites in 1,368 individuals from the population-based Lifelines DEEP (LLD)^5^ and Genome of the Netherlands (GoNL)^6^ cohorts, including LLD_1_ (*n* = 1,054), LLD_2_ (*n* = 237) and GoNL (*n* = 77). In addition, 311 LLD_1_ individuals were followed up after 4 years^7^. For each participant, we had information on the gut microbiome, genetic background and dietary habits. In addition, we assessed whether diet-associated metabolites can be used to predict an individual’s dietary quality score reflecting diet‒disease relationships^8^ and examined whether genetics-associated metabolites can pinpoint dysregulated molecular pathways in complex diseases. Importantly, as potential causal relationships among metabolites, diet and the microbiome remain largely unexplored, metabolites associated with multiple factors offered us an opportunity to infer their underlying causality using Mendelian randomization (MR) and mediation analyses^9^.

## Results

### Untargeted plasma metabolites in Dutch cohorts

**Extended Data Fig. 1:**
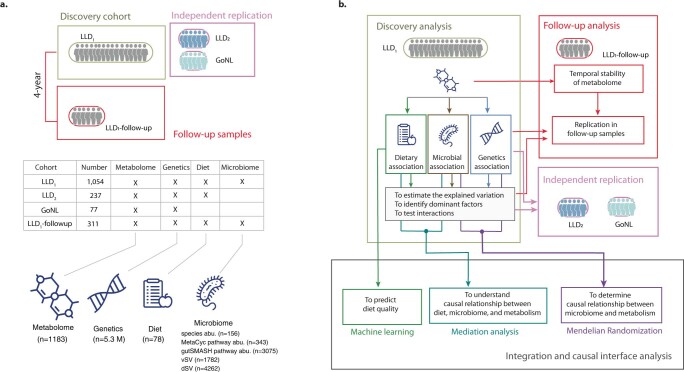
Overview of study cohorts and analysis workflow. **a**. Summary of cohorts and datasets involved in the analyses. **b**. Detailed analysis workflow of the present study.

**Extended Data Fig. 2:**
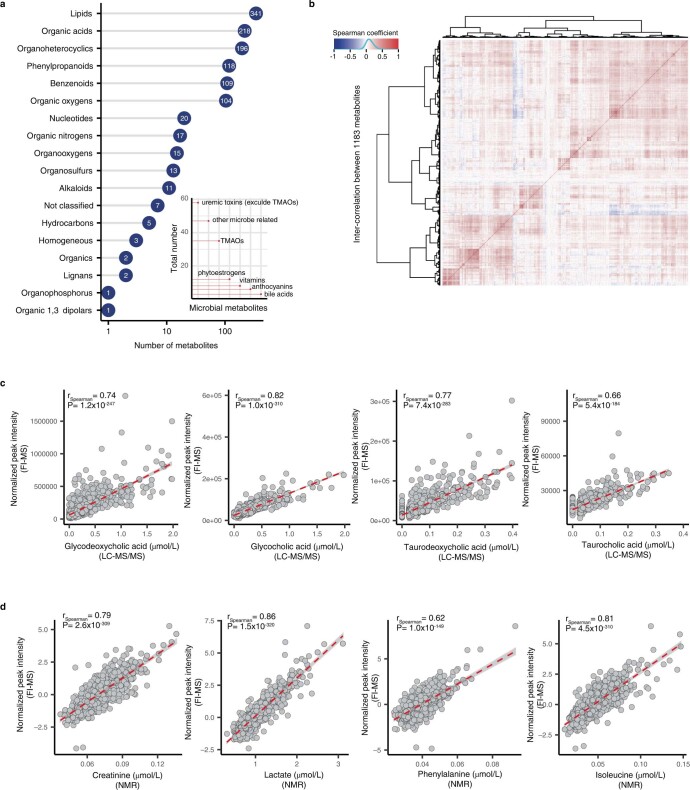
Summary of plasma metabolites. **a**. Number of metabolites per metabolic categories based on annotation in the HMDB database. The x-axis indicates number of metabolites per category. The number of metabolites is also shown. The y-axis indicates metabolic categories. **b**. Inter-correlation between metabolites in the LLD baseline samples. Rows and columns represent metabolites. Color scheme represents the Spearman correlation coefficient. **c**. Comparison of metabolite concentrations between un-targeted FI-MS and LC-MS/MS platforms. The x-axis indicates metabolic abundance determined by LC-MS/MS. The y-axis indicates metabolic abundance determined by FI-MS. Each gray dot represents one sample. The red dashed line is the best fit line of linear regression with 95% confidence interval (CI). Spearman correlation coefficient between two measurements and the corresponding P value (two-sided) are shown. **d**. Comparison of metabolite concentrations between un-targeted FI-MS and NMR platforms. The x-axis indicates metabolic abundance determined by NMR and the y-axis indicates metabolic abundance determined by FI-MS. Each gray dot represents one sample. The red dashed line is the best fit line of linear regression with 95% CI. Spearman correlation coefficients between two measurements and the corresponding P value (two-sided) are shown.

In this study, we examined plasma metabolomes in 1,679 fasting plasma samples from 1,368 individuals from two LLD^5^ sub-cohorts (LLD_1_ and LLD_2_) and the GoNL^6^ cohort (Extended Data Fig. 1 and Supplementary Table 1). The LLD_1_ cohort was the discovery cohort, with information about genetics, diet and the gut microbiome available for 1,054 participants. Moreover, 311 LLD_1_ subjects were followed up 4 years later (LLD_1_ follow-up). We also included two independent replication cohorts: 237 LLD_2_ participants for whom we had genetic and dietary data and 77 GoNL participants for whom only genetic data were available (Extended Data Fig. 1 and Supplementary Table 1). Untargeted metabolomics profiling was done using flow-injection time-of-flight mass spectrometry (FI-MS)^10,11^, which yielded plasma levels of 1,183 metabolites (Supplementary Table 2). These metabolites covered a wide range of lipids, organic acids, phenylpropanoids, benzenoids and other metabolites (Extended Data Fig. 2a). As we observed weak (absolute *r*_Spearman_ < 0.2) correlations among the 1,183 metabolites (Extended Data Fig. 2b), data reduction was not required and, consequently, all metabolites were subjected to subsequent analyses. We validated the identification and quantification of some metabolites (for example, bile acids, creatinine, lactate, phenylalanine and isoleucine) by comparing their abundance levels from FI-MS with those previously determined by liquid chromatography with tandem mass spectrometry (LC-MS/MS)^12^ or NMR^13^ (*r*_Spearman_ > 0.62; Extended Data Fig. 2c,d).

### Factors explaining inter-individual metabolome variations

**Fig. 1:**
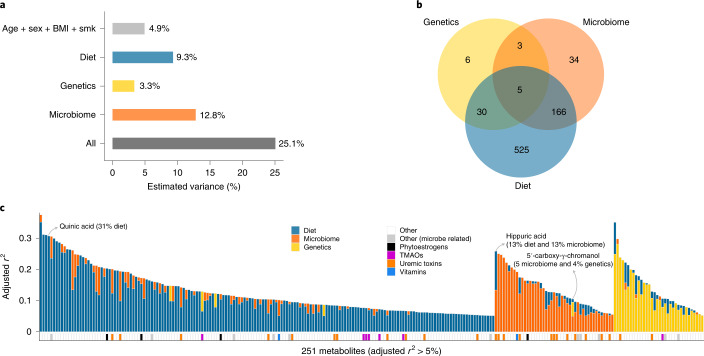
Contributions of genetics, diet and the microbiome to inter-individual variation in the plasma metabolome. **a**, Inter-individual variation in the whole plasma metabolome explained by the indicated factors, estimated using the PERMANOVA method. All, all of the indicated factors combined; smk, smoking status. **b**, Venn diagram indicating the number of metabolites whose inter-individual variation was significantly explained by diet, genetics or the gut microbiome, as estimated using the linear regression method (FDR_*F*-test_ < 0.05). **c**, Inter-individual variations in metabolites explained by diet, genetics or the gut microbiome, as estimated using the linear regression method (the lasso regression method was applied for feature selection) with a significant estimated adjusted *r*^2^ > 5% (FDR_*F*-test_ < 0.05). The blue bars represent dietary contributions to metabolite variations, the yellow bars indicate genetic contributions and the orange bars indicate microbial contributions. The other colors indicate the metabolic categories of metabolites (see legend). The *y* axis indicates the proportion of variation explained. TMAO, trimethylamine *N*-oxide.

To compare the relative importance of diet, genetics and the gut microbiome in explaining inter-individual plasma metabolome variability, we calculated the proportion of variance explained by these three factors for the whole plasma metabolome profile and for the individual metabolites separately. We have detailed information on 78 dietary habits (Supplementary Table 3), 5.3 million human genetic variants and the abundances of 156 species and 343 MetaCyc pathways for each individual of the LLD_1_ cohort. Diet, genetics and the gut microbiome could explain 9.3, 3.3 and 12.8%, respectively, of inter-individual variations in the whole plasma metabolome, without adjusting for covariates (see the Methods section ‘Distance matrix-based variance estimation’; false discovery rate (FDR) < 0.05; Fig. 1a and Supplementary Table 4), whereas intrinsic factors (age, sex and body mass index (BMI)) and smoking collectively explained 4.9% of the variance. Together, these factors explain 25.1% of the variance in the plasma metabolome (Fig. 1a).

Next, we tested for pairwise associations between each metabolite and the dietary variables, genetic variants and microbial taxa. We observed 2,854 associations with dietary habits (Supplementary Table 5), 48 associations with 40 unique genetic variants (metabolite quantitative trait loci (mQTLs); Supplementary Table 6), 1,373 associations with gut bacterial species (Supplementary Table 7) and 2,839 associations with bacterial MetaCyc pathways (Supplementary Table 8) (see the Methods sections ‘Associations with dietary habits’, ‘QTL mapping’ and ‘Microbiome-wide associations’). In total, 769 metabolites were significantly associated with at least one factor (Fig. 1b and Supplementary Tables 5–8). We then performed interaction analysis to assess the role of diet‒microbiome, genetics‒microbiome and diet‒genetics interactions in regulating the human metabolome using an interaction term in the linear model (see the Methods section ‘Interaction analysis’). Among these, 185 metabolites were associated with multiple factors and seven were affected by either genetics–microbiome, genetics–diet or diet–microbiome interactions (Supplementary Table 9).

As interactions were limited, we further assessed the proportion of variance of each metabolite that was explained by these factors using an additive model with the least absolute shrinkage and selection operator (lasso) method (see the Methods section ‘Estimating the variance of individual metabolites’). In general, the inter-individual variations in 733 metabolites could be explained by at least one of the three factors (FDR_*F*-test_ < 0.05; Supplementary Table 10). In detail, dietary habits contributed 0.4‒35% of the variance in 684 metabolites; microbial abundances contributed 0.7‒25% of the variance in 193 metabolites; and genetic variants contributed 3‒28% of the variance in 44 metabolites (adjusted *r*^2^; FDR_*F*-test_ < 0.05; Supplementary Table 10). We also estimated the explained variance of metabolites using Elastic Net^14^, which is designed for highly correlated features, and found that the estimated explained variances were comparable between linear regression and the Elastic Net regression (Supplementary Fig. 1).

We further compared the variance explained by each type of factor (diet, genetics or the microbiome) and assigned the dominant factor for each metabolite if one factor explained more variance than the other two. Inter-individual variations in 610 metabolites were mostly explained by diet, 85 were explained by the gut microbiome and 38 were explained by genetics (Supplementary Table 10). Hereafter, we refer to these as diet-dominant, microbiome-dominant and genetics-dominant metabolites, respectively. The dominant factors of metabolites highlight their origin. For instance, ten out of the 21 diet-dominant metabolites for which diet explained >20% of the variance (FDR_*F*-test_ < 0.05; Supplementary Table 10) were food components based on their annotation in the Human Metabolome Database (HMDB)^15^. Similarly, of the 85 microbiome-dominant metabolites, 23 were annotated in the HMDB as microbiome-related metabolites (including 15 uremic toxins). Furthermore, out of the 38 genetics-dominant metabolites, ten were lipid species and eight were amino acids. Taken together, our analysis highlights that one factor—either dietary, genetic or microbial—can have a dominant effect over the other two in explaining the variances of plasma metabolites, with diet or the microbiome being particularly dominant. However, we also found that the variances in 185 metabolites were significantly attributable to more than one factor (Supplementary Table 10), including six metabolites associated with both genetics and the microbiome and 153 metabolites associated with both diet and the microbiome. For example, genetics and the microbiome explained 4 and 5%, respectively, of the variance in plasma 5′-carboxy-γ-chromanol (Fig. 1c)—a dehydrogenated carboxylate product of 5′-hydroxy-γ-tocopherol^16^ that may reduce cancer and cardiovascular risk^17^. Another example is hippuric acid—a uremic toxin that can be produced by bacterial conversion of dietary proteins^18^, with 13% of its variance explained by diet and 13% explained by the microbiome (Fig. 1c).

### Temporal variability of the metabolites over time

**Fig. 2:**
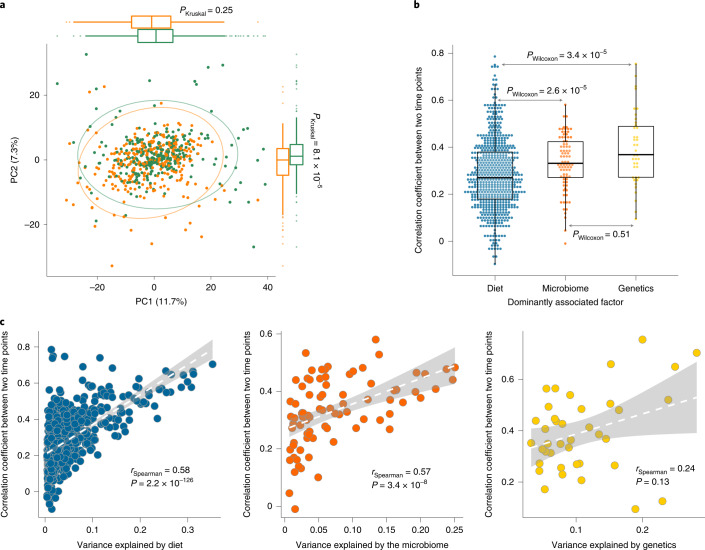
Temporal stability of plasma metabolites. **a**, Principal component analysis of metabolite levels at two time points (Euclidean dissimilarity). The green dots indicate baseline samples and the orange dots indicate follow-up samples (*n* = 311 biologically independent samples). The Kruskal–Wallis test (two sided) was used to check differences between baseline and follow-up. **b**, Temporal stability of metabolites stratified by the dominantly associated factor for each metabolite. The Wilcoxon test (two sided) was used to check the differences between groups. Each dot represents one metabolite. The *y* axis indicates the Spearman correlation coefficient of abundances of each metabolite between two time points (*n* = 311 biologically independent samples). In **a** and **b**, the box plots show the median and first and third quartiles (25th and 75th percentiles) of the first and second principal components (**a**) or correlation coefficients (**b**); the upper and lower whiskers extend to the largest and smallest value no further than 1.5× the interquartile range (IQR), respectively; and outliers are plotted individually. **c**, Correlation between metabolite stability and the metabolite variance explained by diet (left), genetics (middle) and the microbiome (right). The *x* axis indicates the inter-individual variation explained by each factor and the *y* axis indicates the Spearman correlation coefficient (two sided) of abundances of each metabolite between the two time points. The dashed white lines show the best fit and the gray shading represents the 95% confidence interval (CI) (*n* = 311 biologically independent samples).

**Extended Data Fig. 3:**
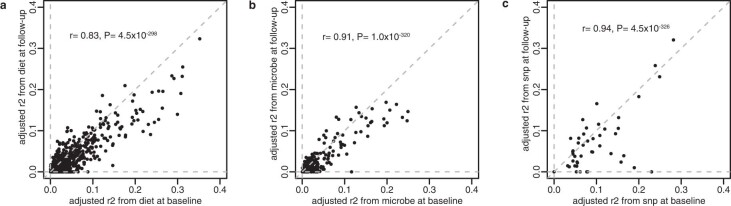
Comparison of the proportion of metabolite variation explained by diet, microbiome and genetics at two time points. **a**. Proportion of the variation explained by diet. **b**. Proportion of the variation explained by microbiome. **c**. Proportion of the variation explained by genetics. Each dot represents a metabolite. The x-axis indicates explained variation at baseline and the y-axis indicates explained variation at follow-up. Similarity between the two time points was assessed using Spearman correlation (two-sided).

Temporal changes in plasma metabolites can reflect changes in an individual’s diet, gut microbiome and health status. When assessing the plasma metabolome in the 311 LLD_1_ follow-up samples, we indeed observed a significant shift in the plasma metabolome, with a significant difference in the second principal component (*P*_PC1 paired Wilcoxon_ = 0.1 and *P*_PC2 paired Wilcoxon_ = 1.3 × 10^−5^; Fig. 2a). Baseline genetics, diet and microbiome, together with age, sex and BMI, could explain 59.4% of the variance in the follow-up plasma metabolome (*P*_PERMANOVA_ = 0.004) (Supplementary Fig. 2). We also observed that temporal stability can vary substantially between different metabolites (see the Methods section ‘Temporal consistency of individual metabolites’; Supplementary Table 11). Previously, we had assessed the changes in the gut microbiome in the LLD_1_ follow-up cohort and linked these to changes in the plasma metabolome^7^. Here, we further checked the temporal variability of the plasma metabolome and assessed the stability of diet-, microbiome- and genetics-dominant metabolites over time. Interestingly, the temporal correlation of the microbiome-dominant metabolites was similar to that of the genetics-dominant metabolites (*P*_Wilcoxon_ = 0.51; Fig. 2b), whereas the temporal correlation between diet-dominant metabolites was significantly lower than between microbiome- and genetics-dominant metabolites (*P*_Wilcoxon_ < 3.4 × 10^−5^; Fig. 2b). However, the dominant dietary, microbial and genetic factors identified at baseline also explained similar variance in metabolic levels in the follow-up samples (Extended Data Fig. 3 and Supplementary Table 10). Our data also revealed a positive correlation between stability and the amount of variance that could be explained: the more variance explained, the more stable a metabolite is over time (Fig. 2c). For a few metabolites, we could not replicate the variance explained at baseline at the second time point, and these metabolites also showed weak or no correlation in their abundances between the two time points. For example, *N*-acetylgalactosamine showed very weak correlation between the two time points (*r* = 0.13; *P* = 0.02), and its genetic association was not replicated at the second time point.

Having established the variances in metabolites explained by diet, genetics and the gut microbiome and the dominant factors that explained most of this variance, we focused on detailing specific associations and on the potential implications of our findings for assessing diet quality and improving our understanding of the genetic risk of complex diseases and the interaction and causality relationships among diet, the microbiome, genetics and metabolism.

### The metabolome reflects the diet quality score

**Fig. 3:**
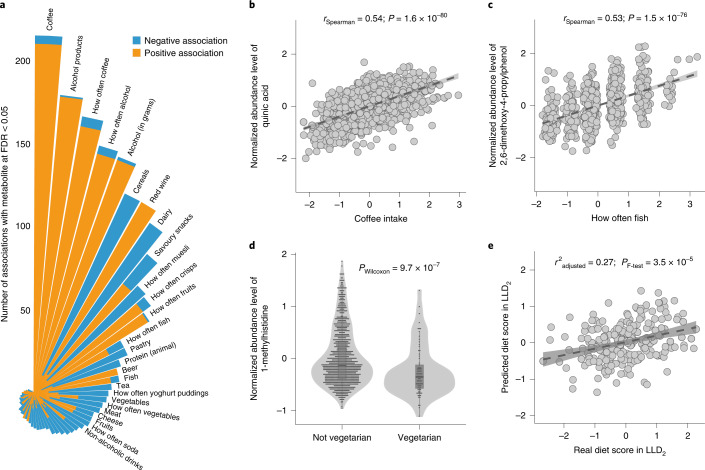
Associations between dietary habits and plasma metabolites. **a**, Summary of the associations between diet and metabolites. The bars represent dietary habits, with the bar order sorted by the number of significant associations. Association directions are colored differently: orange indicates a positive association, whereas blue indicates a negative association. The length of each bar indicates the number of significant associations at FDR < 0.05 (Spearman; two sided). **b**, Association between plasma quinic acid levels and coffee intake. The *x* and *y* axes indicate residuals of coffee intake and the metabolic abundance after correcting for covariates, respectively (*n* = 1,054 biologically independent samples). **c**, Association between plasma 2,6-dimethoxy-4-propylphenol levels and fish intake frequency (*n* = 1,054 biologically independent samples). The *x* and *y* axes refer to residuals of fish intake and metabolic abundance after correcting for covariates, respectively. **d**, Differential plasma levels of 1-methylhistidine between vegetarians and non-vegetarians (*n* = 1,054 biologically independent samples). The *y* axis indicates normalized residuals of metabolic abundance. The *P* value from the Wilcoxon test (two sided) is shown. The box plots show the median and first and third quartiles (25th and 75th percentiles) of the metabolite levels. The upper and lower whiskers extend to the largest and smallest value no further than 1.5× the IQR, respectively. Outliers are plotted individually. **e**, Association between the diet quality score predicted by the plasma metabolome (*y* axis) and the diet quality score assessed by the FFQ (*x* axis) (*n* = 237 biologically independent samples). In **b**, **c** and **e**, each gray dot represents one sample, the dark gray dashed line shows the linear regression line and the gray shading represents the 95% CI. In **b** and **c**, the association strength was assessed using Spearman correlation (two sided; the correlation coefficient and *P* value are reported) and in **e**, the prediction performance was assessed with linear regression (*F*-test; two sided; the adjusted *r*^2^ value and *P* value are reported).

We observed 2,854 significant associations (FDR_Spearman_ < 0.05) between 74 dietary factors and 726 metabolites (Fig. 3a and Supplementary Table 5; see the Methods section ‘Lifelines diet quality score prediction’). Associations with food-specific metabolites can, in theory, be used to verify food questionnaire data. For instance, the strongest association we observed was between quinic acid levels and coffee intake (*r*_Spearman_ = 0.54; *P* = 1.6 × 10^−80^; Fig. 3b). Quinic acid is found in a wide variety of different plants but has a particularly high concentration in coffee. Another example is 2,6-dimethoxy-4-propylphenol, which was strongly associated with fish intake (*r*_Spearman_ = 0.53; *P* = 1.5 × 10^−76^; Fig. 3c). This association is expected as this compound is particularly present in smoked fish according to HMDB annotation^15^. In addition, we also detected associations between dietary factors and metabolic biomarkers of some diseases. For example, 1-methylhistidine is a biomarker for cardiometabolic diseases including heart failure^19^ that is enriched in meat, and we observed significant associations between 1-methylhistidine and meat (*r*_Spearman_ = 0.12; *P* = 7.2 × 10^−5^) and fish intake (*r*_Spearman_ = 0.11; *P* = 3.1 × 10^−4^) as well as a lower level of 1-methylhistidine in vegetarians (*r*_Spearman_ = −0.15; *P* = 9.7 × 10^−7^; Fig. 3d).

Given the relationship between diet, metabolism and human health, we wondered whether the plasma metabolome could predict diet quality. For each of the Lifelines participants, we constructed a Lifelines Diet Score based on food frequency questionnaire (FFQ) data that reflected the relative diet quality based on diet‒disease relationships^8^. To build a metabolic model to predict an individual’s diet quality, we used LLD_1_ as the training set and LLD_2_ as the validation set. The resulting metabolic model included 76 metabolites, 51 of which were dominantly associated with diet. The diet score predicted by metabolites showed a significant association with the real diet score assessed by the FFQ in the validation set (*r*^2^_adjusted_ = 0.27; *P*_*F*-test_ = 3.5 × 10^−5^; Fig. 3e). We also tested four other dietary scores (the Alternate Mediterranean Diet Score^20^, Healthy Eating Index (HEI)^21^, Protein Score^22^ and Modified Mediterranean Diet Score^23^) and found that the HEI predicted by plasma metabolites was also significantly associated with the FFQ-based HEI (*r*^2^_adjusted_ = 0.23; *P*_*F*-test_ = 6.5 × 10^−5^; Supplementary Table 12).

### Genetic associations of plasma metabolites

**Fig. 4:**
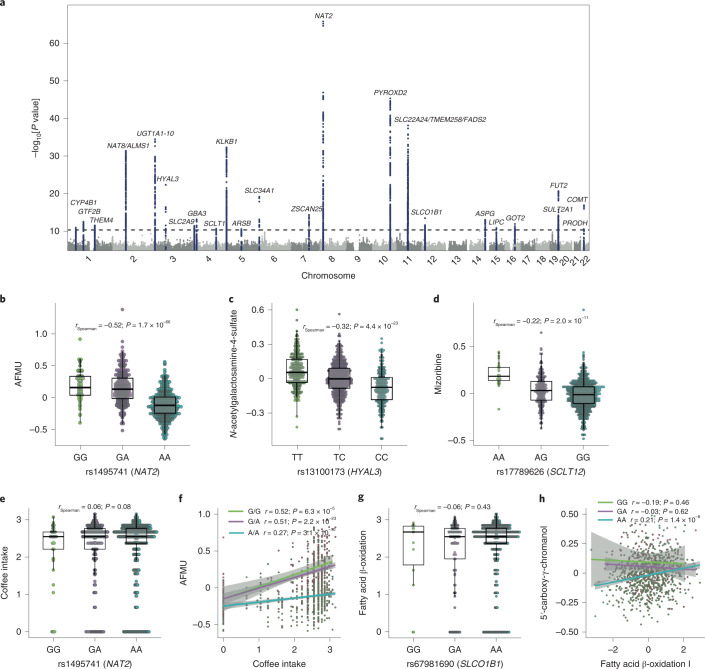
Genetic associations of plasma metabolites. **a**, Manhattan plot showing 48 independent mQTLs identified linking 44 metabolites and 40 genetic variants with *P* < 4.2 × 10^−11^ (Spearman; two sided). Representative genes for the SNPs with significant mQTLs are labeled. **b**, Association between a tag SNP (rs1495741) of the *NAT2* gene and plasma AFMU levels. **c**, Association between a SNP (rs13100173) within the *HYAL3* gene and plasma levels of *N*-acetylgalactosamine-4-sulfate. **d**, Association between a tag SNP (rs17789626) of the *SCLT1* gene and plasma mizoribine levels. **e**, Differences in coffee intake between participants with different genotypes at rs1495741. **f**, Correlations between coffee intake and AFMU in participants with different genotypes at rs1495741. **g**, Differences in bacterial fatty acid β-oxidation pathway abundance in participants with different genotypes at rs67981690. **h**, Correlations between bacterial fatty acid β-oxidation pathway abundance and 5′-carboxy-γ-chromanol in participants with different genotypes at rs67981690. In **b**–**e** and **g**, the *x* axis indicates the genotype of the corresponding SNP and the *y* axis indicates normalized residuals of the corresponding metabolic abundance (*n* = 927 biologically independent samples). Each dot represents one sample. The box plots show the median and first and third quartiles (25th and 75th percentiles) of the metabolite levels. The upper and lower whiskers extend to the largest and smallest value no further than 1.5× the IQR, respectively. Outliers are plotted individually. The association strength is shown by the Spearman correlation coefficient and corresponding *P* value (two sided). In **f** and **h**, the *x* axis indicates the normalized abundance of coffee intake (**f**) or the bacterial fatty acid β-oxidation pathway (**h**) and the *y* axis indicates the normalized residuals of the corresponding metabolic abundance. Each dot represents one sample (*n* = 927 biologically independent samples). The lines indicate linear regressions for each genotype group separately. Areas with light gray shading indicate the 95% CI of the linear regression lines. The association strength per genotype is shown by the Spearman correlation and the corresponding *P* value (two sided).

**Extended Data Fig. 4:**
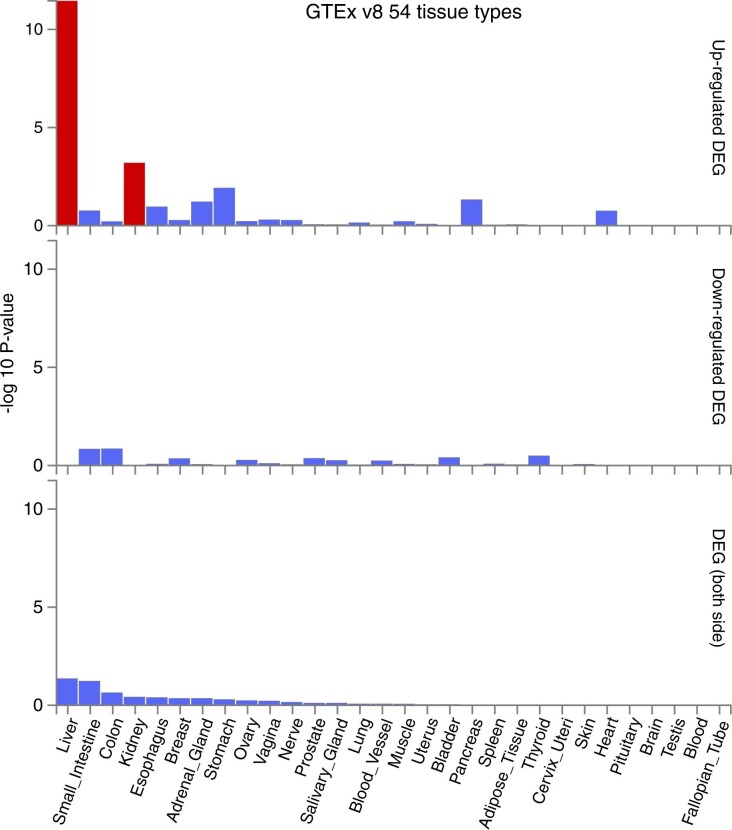
Tissue-specific gene expression analysis with FUMA. All significant mQTLs observed in LLD_1_ were used to check the enrichment of differentially expressed gene (DEG) sets in a specific tissue compared to all other tissue types based on GTEx (version 8). Red bars represent liver and kidney. Blue bars represent other tissues. The x-axis is the different tissue type and the y-axis indicates the significance of the differential abundance of the gene in one tissue compared to other tissue types in terms of -log10 transformed P value (Fisher’s exact test, two-sided).

Genetic associations of plasma metabolites may provide functional insights into the etiologies of complex diseases. After correcting for the first two genetic principal components, age, sex, BMI, smoking, 78 dietary habits, 40 diseases and 44 medications, QTL mapping in LLD_1_ identified 48 study-wide, independent genetic associations between 44 metabolites and 40 single-nucleotide polymorphisms (SNPs) (*P*_Spearman_ < 4.2 × 10^−11^; clumping *r*^2^ = 0.05; clumping window = 500 kilobases (kb); Fig. 4a and Supplementary Table 6). All 48 genetic associations were replicated in either LLD_1_ follow-up or the two independent replication datasets (LLD_2_ and GoNL; Supplementary Fig. 3 and Supplementary Table 6). We also assessed the impact of physical activity, as assessed by questionnaires^24^, on the genetics association of metabolism, but found its influence to be negligible (Supplementary Fig. 4). Functional mapping and annotation (FUMA) of genome-wide association studies (GWAS)^25^ analysis revealed that the identified mQTLs were enriched in genes expressed in the liver and kidney (Extended Data Fig. 4) and related to metabolic phenotypes (Supplementary Table 6).

The strongest association we found was between the caffeine metabolite 5-acetylamino-6-formylamino-3-methyluracil (AFMU) and SNP rs1495741 near the *N*-acetyltransferase 2 (*NAT2*) gene (*r*_Spearman_ = −0.52; *P* = 1.7 × 10^−66^; Fig. 4b), which showed strong linkage disequilibrium (*r*^2^ = 0.98) with a SNP, rs35246381, that was recently reported to be associated with urinary AFMU^26^. AFMU is a direct product of NAT2 activity and has been associated with bladder cancer risk^27^. Interestingly, the plasma level of AFMU was associated not only with coffee intake (*r*_Spearman_ = 0.29; *P* = 9.2 × 10^−22^; Supplementary Table 5) and the genotype of rs1495741, but also with their interactions (Supplementary Table 9). Individuals with a homologous AA genotype had a similar level of coffee intake, but their correlation between coffee intake and plasma AFMU level was significantly lower compared with individuals with GG and GA genotypes (Fig. 4e,f).

Pleotropic mQTL effects were also observed at several loci, including *SLCO1B1*, *FADS2*, *KLKB1* and *PYROXD2* (Supplementary Table 6). For example, three associations (related to three metabolites, two of them lipids) were observed for two SNPs (rs67981690 and rs4149067; linkage disequilibrium *r*^2^ = 0.72 in Northern Europeans from Utah) in *SLCO1B1*, which encodes the solute carrier organic anion transporter family member 1B1. Expression of the SLCO1B1 protein is specific to the liver, where this transporter is involved in the transport of various endogenous compounds and drugs, including statins^28^, from blood into the liver. The SLCO1B1 locus has also been linked to plasma levels of fatty acids and to statin-induced myopathy^29^. Furthermore, we detected a genetics–microbiome interaction between rs67981690 and microbial fatty acid oxidation pathways in regulating plasma levels of 5′-carboxy-γ-chromanol (*P* = 1.5 × 10^−3^), where the association of the bacterial fatty acid oxidation pathway with plasma levels of 5′-carboxy-γ-chromanol was dependent on the genotype of rs67981690 (Fig. 4g,h).

To identify novel mQTLs, we performed a systematic search of all published mQTL studies from 2008 onwards (Supplementary Table 13). This approach identified three novel mQTLs in our datasets (Supplementary Table 13) that were either not located close to previously reported mQTLs (distance > 1,000 kb) or not in linkage disequilibrium (*r*^2^ < 0.05). The first two novel SNPs—rs13100173 at *HYAL3* and rs11741352 at *ARSB*—were associated with *N*-acetylgalactosamine-4-sulfate (Fig. 4c,d), which is associated with mucopolysaccharidosis^30^. Interestingly, *N*-acetylgalactosamine-4-sulfate can bind to HYAL proteins (HYAL1, HYAL2, HYAL3 and HYAL4), suggesting that mQTLs can also pinpoint potential metabolite–protein interactions. The third novel mQTL was rs17789626 at *SCLT1*, which was associated with mizoribine—a compound used to treat nephrotic syndrome^31^.

### A causal role for the microbiome in determining metabolites

We established 4,212 associations between 208 metabolites and 314 microbial factors (114 species and 200 MetaCyc pathways) (FDR_LLD1_ < 0.05; *P*_LLD1 follow-up_ < 0.05; Supplementary Tables 7 and 8). Interestingly, many of the metabolites that were associated with microbial species and MetaCyc pathways are also known to be gut microbiome related based on their HMDB annotations^15^. For instance, we observed 919 associations with 25 uremic toxins, 142 associations with thiamine (vitamin B1) and 117 associations with five phytoestrogens (FDR < 0.05; Supplementary Tables 7 and 8). Uremic toxins and thiamine have been shown to be related to various diseases, including chronic kidney disease and cardiovascular diseases^32,33^. Phytoestrogens are a class of plant-derived polyphenolic compounds that can be transformed by gut microbiota into metabolites that promote the host’s metabolism and immune system^33,34^.

**Fig. 5:**
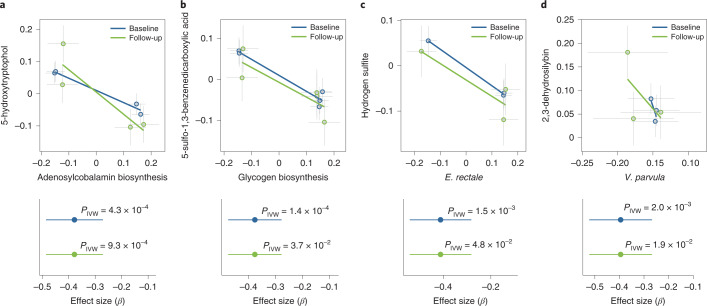
Causal relationships between microbiomes and plasma metabolites as assessed by MR analysis. **a**, Analysis of the association between adenosylcobalamin biosynthesis pathway abundance and 5-hydroxytryptophol levels. **b**, Glycogen biosynthesis pathway abundance versus 5-sulfo-1,3-benzenedicarboxylic acid levels. **c**, *E. rectale* abundance versus hydrogen sulfite levels. **d**, *Veillonella parvula* abundance versus 2,3-dehydrosilybin levels. In the top panels of **a**–**d**, the *x* axis shows the SNP exposure effect, and the *y* axis shows the SNP outcome effect and each dot represents a SNP. Error bars represent the s.e. of each effect size. The bottom panels of **a**–**d**, show the MR effect size (center dot) and 95% CI for the baseline (blue) and follow-up (green) datasets of the LLD_1_ cohort, estimated with the IVW MR approach (two sided) (*n* = 927 biologically independent samples at baseline and *n* = 311 biologically independent samples at follow-up).

**Extended Data Fig. 5:**
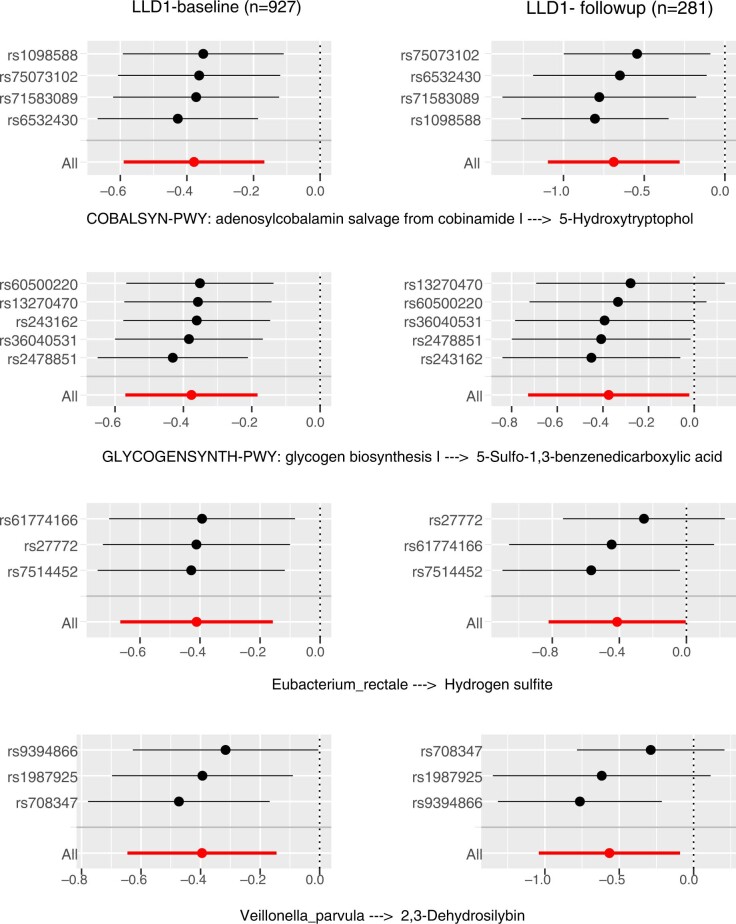
Leave-one-out sensitivity analysis for significant MR linkages. Forest plots of MR leave-one-out sensitivity results (IVW method) for four significant bi-directional MR linkages in LLD_1_ and LLD_1_-follow-up. Dots represent the estimated effect size. Bars represent 95% confidence intervals. The x-axis indicates effect size of MR.

To assess whether gut microbiome composition causally contributes to plasma metabolite levels, we carried out bi-directional MR analyses (see the Methods section ‘Bi-directional MR analysis’). Here, we focused on the 37 microbial features that were associated with at least three independent genetic variants at *P* < 1 × 10^−5^ and with 45 metabolites (Supplementary Table 14). At FDR < 0.05 (corresponding to *P* = 2 × 10^−3^ obtained from the inverse variance weighted (IVW) test)^35^, we observed four potential causal relationships at baseline that could also be found in the follow-up in the microbiomes to metabolites direction (Fig. 5a–d and Supplementary Tables 15 and 16) but not in the opposite direction (Supplementary Table 17), and these outcomes were maintained following weighted median testing (*P* < 0.03; Supplementary Fig. 5). To ensure that the data followed MR assumptions, we performed several sensitivity analyses, including checking for horizontal pleiotropy (MR-Egger^36^ intercept *P* > 0.05; Supplementary Table 15) and heterogeneity (Cochran’s *Q* test *P* > 0.05; Supplementary Table 15) and leave-one-out analysis (Extended Data Fig. 5). We did not use causal estimates derived using the MR-Egger method to filter the results, as its power to detect causality is known to be low^36^. These sensitivity checks further confirmed the reliability of these four MR causal estimates.

We further found that increased abundance of microbial adenosylcobalamin biosynthesis (coenzyme B12) was associated with reduced plasma levels of 5-hydroxytryptophol (Fig. 5a)—a uremic toxin related to Parkinson’s disease^37^. We also found that plasma hydrogen sulfite levels were related to *Eubacterium rectale* (Fig. 5c)—a core gut commensal species^38^ that is highly prevalent (presence rate = 97%) and abundant (mean abundance = 8.5%) in both our cohorts and in other populations^39–41^. As a strict anaerobe, *E. rectale* promotes the host’s intestinal health by producing butyrate and other short-chain fatty acids from non-digestible fibers^42^, and a reduced abundance of this species has been observed in subjects with inflammatory bowel disease^39,43^ and colorectal cancer^44^ compared with healthy controls. As a toxin, hydrogen sulfite interferes with the nervous system, cardiovascular functions, inflammatory processes and the gastrointestinal and renal system^45^. Our results thus reveal a potential new beneficial effect of *E. rectale*.

**Extended Data Fig. 6:**
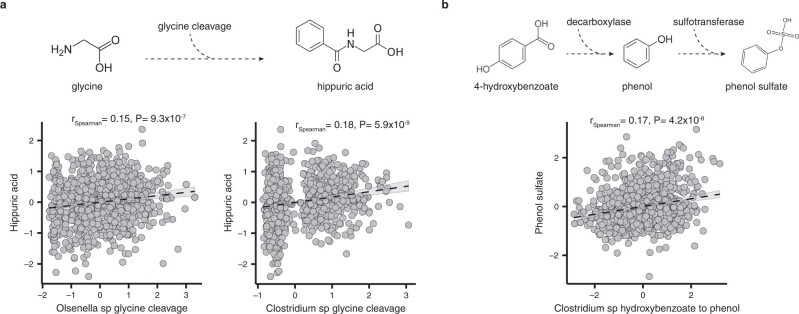
Metabolites associated with gutSMASH pathways. **a**. Correlation between the microbial glycine cleavage pathway and plasma hippuric acid levels. **b**. Correlation between the microbial hydroxybenzoate to phenol pathway and plasma phenol sulfate levels. The upper part of each panel indicates the chemical transformation of the gutSMASH pathways. The lower part indicates the correlation between the pathway abundance (x-axis) and the plasma level of the corresponding metabolite (y-axis). Each dot represents one sample. The dark dashed line is the best fitted line of linear regression with 95% CI. Spearman correlation coefficient and the corresponding P value (two-sided) are also shown.

To further investigate the metabolic potential of individual bacterial species, we applied newly developed pipelines to identify microbial primary metabolic gene clusters (gutSMASH pathways)^46^ and microbial genomic structural variants (SVs)^47^. These two tools profile microbial genomic entities that are implicated in metabolic functions. By associating 1,183 metabolites with 3,075 gutSMASH pathways and 6,044 SVs (1,782 variable SVs (vSVs) and 4,262 deletion SVs (dSVs); see Methods), we observed 23,662 associations with gutSMASH pathways and 790 associations with bacterial SVs (FDR_LLD1_ < 0.05; *P*_LLD1 follow-up_ < 0.05; Supplementary Tables 18–20). These associations connect the genetically encoded functions of microbes with metabolites, thereby providing putative mechanistic information underlying the functional output of the gut microbiome. In one example, we observed that the microbial uremic toxin biosynthesis pathways, including the glycine cleavage pathway (in *Olsenella* and *Clostridium* species) and the hydroxybenzoate-to-phenol pathway (in *Clostridium* species) responsible for hippuric acid and phenol sulfate biosynthesis, were associated with the hippuric acid (*Olsenella* species: *r*_Spearman_ = 0.15; *P* = 9.3 × 10^−7^; *Clostridium* species: *r*_Spearman_ = 0.18; *P* = 5.9 × 10^−9^) and phenol sulfate (*r*_Spearman_ = 0.17; *P* = 4.2 × 10^−8^; Extended Data Fig. 6a) levels measured in plasma, respectively (FDR_LLD1_ < 0.05 and *P*_LLD1 follow-up_ < 0.05; Extended Data Fig. 6b).

### Diet‒microbiome mediation effects in the control of metabolites

**Fig. 6:**
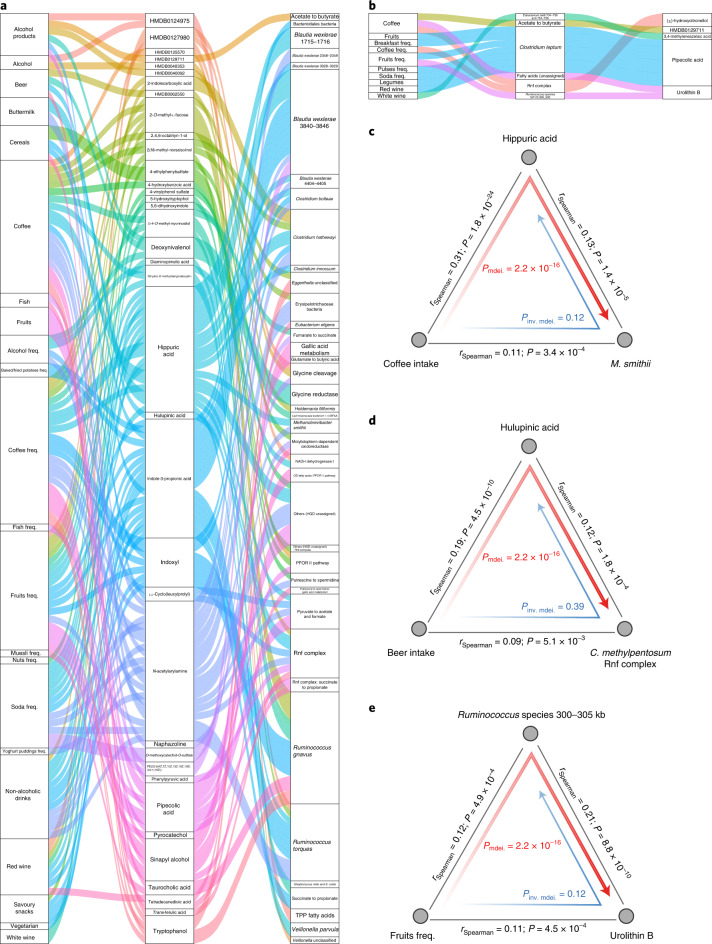
Mediation analysis identifies linkages between the gut microbiome, metabolites and dietary habits. **a**, Parallel coordinates chart showing the 133 mediation effects of plasma metabolites that were significant at FDR < 0.05. Shown are dietary habits (left), plasma metabolites (middle) and microbial factors (right). The curved lines connecting the panels indicate the mediation effects, with colors corresponding to different metabolites. freq., frequency; PFOR, pyruvate:ferredoxin oxidoreductase; OD, oxidative decarboxylation; HGD, 2-hydroxyglutaryl-CoA dehydratase; TPP, thiamine pyrophosphate. **b**, Parallel coordinates chart showing the 13 mediation effects of the microbiome that were significant at FDR < 0.05. Shown are dietary habits (left), microbial factors (middle) and plasma metabolites (right). For the microbial factors column, number ranges represent the genomic location of microbial structure variations (SVs) in kilobyte unit, and colons represent the detailed annotation of certain gutSMASH pathway. **c**, Analysis of the effect of coffee intake on the abundance of *M. smithii* as mediated by hippuric acid. **d**, Analysis of the effect of beer intake on the *C. methylpentosum* Rnf complex pathway as mediated by hulupinic acid. **e**, Analysis of the effect of fruit intake on urolithin B in plasma as mediated by a vSV in *Ruminococcus* species (300‒305 kb). In **c**–**e**, the gray lines indicate the associations between the two factors, with corresponding Spearman coefficients and *P* values (two sided). Direct mediation is shown by a red arrow and reverse mediation is shown by a blue arrow. Corresponding *P* values from mediation analysis (two sided) are shown. inv., inverse; mdei., mediation.

Next, we carried out a mediation analysis to investigate the links between diet, the microbiome and metabolites. For 675 microbial features that were associated with both dietary habits and metabolites (FDR < 0.05), we applied bi-directional mediation analysis to evaluate the effects of microbiome and metabolites for diet (see the Methods section ‘Bi-directional mediation analysis’). This approach established 146 mediation linkages: 133 for the dietary impact on the microbiome through metabolites and 13 for the dietary impact on metabolites through the microbiome (FDR_mediation_ < 0.05 and *P*_inverse-mediation_ > 0.05; Fig. 6a,b and Supplementary Table 21). Most of these linkages were related to the impact of coffee and alcohol on microbial metabolic functionalities (Fig. 6a).

Coffee contains various phenolic compounds that can be converted to hippuric acid by colonic microflora^48^. Hippuric acid is an acyl glycine that is associated with phenylketonuria, propionic acidemia and tyrosinemia^49^. We observed that hippuric acid can mediate the impact of drinking coffee on *Methanobrevibacter smithii* abundance (*P*_mediation_ = 2.2 × 10^−16^; Fig. 6c). We also observed that hulupinic acid, which is commonly detected in alcoholic drinks, can mediate the impact of beer consumption on the *Clostridium methylpentosum* ferredoxin:NAD^+^ oxidoreductase (Rnf) complex (*P*_mediation_ = 2.2 × 10^−16^; Fig. 6d)—an important membrane protein in driving the ATP synthesis essential for all bacterial metabolic activities^50^.

Of the dietary impacts on metabolites through the microbiome (Fig. 6b and Supplementary Table 21), one interesting example is a *Ruminococcus* species vSV (300‒305 kb) that encodes an ATPase responsible for transmembrane transport of various substrates^51^. This *Ruminococcus* species vSV mediated the effect of fruit consumption on plasma levels of urolithin B (*P*_mediation_ = 2.2 × 10^−16^; Fig. 6e). Urolithin B is a gut microbiota metabolite that protects against myocardial ischemia/reperfusion injury via the p62/Keap1/Nrf2 signaling pathway^52^. Taken together, our data provide potential mechanistic underpinnings for diet‒metabolite and diet‒microbiome relationships.

## Discussion

By generating fasting plasma profiles of 1,183 metabolites in 1,679 samples from 1,368 individuals (311 with 4-year follow-up data) for whom we also have extensive dietary records, genetics and gut microbiome data, we carried out systematic diet, genetics and microbiome association analyses. Our results show that diet and the gut microbiome play a more dominant role than genetics in explaining inter-individual variability in metabolism, and the more variance that was explained in a metabolite, the more stable that metabolite was over time.

Dietary components are fundamental resources for the plasma metabolome, and a recent study illustrated that an individual’s dietary habits can predict the levels of specific metabolites present in plasma^3^, highlighting that the plasma metabolome mirrors personal dietary habits. Nevertheless, it remained to be established whether it was possible to assess an individual’s diet quality score based on their plasma metabolome. Using a machine learning-based prediction model, we showed that diet quality estimated by an individual’s plasma metabolome showed a significant correlation with diet quality estimated by the FFQ, suggesting that the plasma metabolome to some extent reflects diet quality.

Dietary components serve as substrates in gut microbial metabolic pathways, leading to the formation of a series of metabolites that can be absorbed from the intestine into the host’s circulation. Although earlier studies had linked gut microbial taxonomic abundances to plasma metabolites^3,4,13,41,53^, these investigations did not capture the specific microbial enzymes responsible for metabolite generation, even though this information is required to connect associated links to underlying molecular mechanisms^54^. Using gutSMASH and microbial SVs, we identified putative metabolic functionalities for previously unannotated microbial genetic sequences. In addition, through bi-directional mediation analysis, we identified hundreds of mediation linkages that provide insight into diet‒microbiome interactions in human metabolic health, as illustrated by several metabolites (for example, phenol and pipecolic acid) that have previously been related to cardiometabolic and kidney diseases^32^. Notably, these mediation linkages mainly show that the impact of diet composition on the microbiome can be mediated by metabolites, highlighting the pronounced selective power of dietary habits in shaping the gut microbiome. Nevertheless, as these results are mainly based on observational data, interpretation of such associations should be made with caution, and future intervention and experimental studies that focus on specific diet and microbial genomic capacities are essential to confirm causality.

Apart from diet and the gut microbiome, human genetics also acts as a potential determinant of the plasma metabolome. With this metabolome dataset, we not only replicated previously reported mQTLs, but we also identified three mQTLs involving three loci not previously known to be associated with any metabolites. The mQTLs we characterized could be linked to cardiometabolic and chronic kidney diseases, as illustrated by the tissue-specific gene expression analysis and pleotropic mQTL effects. We also used genetic variants as instruments in MR to infer causal relationships between the gut microbiome and metabolites. This analysis showed that the microbiome may causally contribute to the levels of toxins (hydrogen sulfite and 5-hydroxytryptophol) related to chronic kidney disease and cardiometabolic diseases^32^. The causal relationships between microbiomes and metabolites that we have established thus reveal potential metabolic functionalities of gut microbes that impact on human health.

We acknowledge several limitations in our study. Untargeted plasma metabolome was profiled using FI-MS without compound separation using liquid chromatography columns, and no genuine standards were used. Although the abundances of a few metabolites were well validated using the LC-MS/MS or NMR platforms, identification and quantification of mass peaks using the FI-MS approach is still generally less accurate than in the classical LC-MS/MS platform. We systematically investigated the contributions of genetics, diet and the microbiome to inter-individual metabolome variations and replicated the explained variance in two independent metabolic profile assessments of the same cohort performed 4 years apart. However, overfitting may still have been an issue and could potentially have biased the conclusions. Our findings should be further replicated using an independent cohort for which similarly extensive datasets on the metabolome, genetics, the microbiome, diet, disease and medication are also available. However, it is challenging to obtain such cohorts. We included as many participants as possible for the replication, including two independent sets of individuals from the LLD_2_ and GoNL cohorts. Nonetheless, our study was still underpowered. At the observed effect size in the discovery set and at the *P* < 0.05 level, we have 80% power to replicate the findings for 100% of the genetic associations, but only 60% power for the microbial associations. In addition, causal relationships between the microbiome and metabolites were based on one-sample MR; replication in independent cohorts with larger sample sizes and two-sample MR analysis may further strengthen those observations and better establish possible biological significance. Finally, the LLD cohort was comprised of Dutch participants of Caucasian ethnicity from the northern region of the Netherlands. It is thus possible that the LLD results are biased towards a region-specific microbial background constrained by host genetics and local environmental exposures. We primarily focused on biologically plausible mechanisms by integrating different layers of omics to provide mechanistic hypotheses, and experimental validation is thus warranted.

Taken together, the dietary, genetic and microbial associations with plasma metabolites and the causal and mediation linkages that we report here provide a comprehensive resource that can guide follow-up studies aimed at designing preventive and therapeutic strategies for human metabolic health.

## Methods

### Study cohorts

The LLD cohort (*n* = 1,500) is a sub-cohort of the large prospective Lifelines cohort study from the north of the Netherlands^5,55^. The cohort is 58% female and 42% male, with a mean age of 45.04 years (s.d. = 13.60). The mean BMI is 25.26 (s.d. = 4.18) and 12% of participants are obese (BMI > 30)^5^. All Lifelines participants signed an informed consent form before sample collection. The ethics review board of the University Medical Center Groningen has approved the study with reference number M12.113965. For this study, we involved 1,054 out of 1,500 LLD participants (LLD_1_) for whom detailed dietary habit, stool microbiome and plasma untargeted metabolomics information is available. After removing relatives and genetic outliers, 927 individuals were subjected to genetics analysis, and for 311 of them we also have 4-year follow-up metabolome data (Supplementary Table 1). We further included several replication cohorts: the GoNL cohort (*n* = 77)^56^ and the remaining set of LLD participants for whom we have genetics and plasma metabolome data but not microbiome data (*n* = 237; LLD_2_) (Fig. 1a and Supplementary Table 1).

### Data generation and preprocessing

#### Plasma metabolome

Plasma samples of study participants were collected and frozen at −80 °C with ethylenediaminetetraacetic acid. During extraction, plasma samples were thawed on ice, vortexed and spun down. Then, 20 µl plasma was combined with 180 µl 80% methanol and vortexed for 15 s. The samples were then incubated at 4 °C for 1 h to precipitate the proteins and then spun for 30 min at 3,200 RCF.

Untargeted metabolic profiling of fasting plasma samples was conducted at General Metabolics using FI-MS on an Agilent 6550 Q-TOF system^10,11^. In brief, the instrument was set to scan in full mass spectrometry at 1.4 Hz in negative ionization, 4 GHz High Res Mode and from 50–1,000 *m/z*. The solvent was 60:40 isopropanol:water supplemented with 1 mM NH_4_F at pH 9.0, as well as 10 nM hexakis(1H,1H,3H-tetrafluoropropoxy)phosphazene and 80 nM taurocholic acid for online mass calibration. Then, 100 µl of samples were injected into the ionization source in a random order. Data were acquired in profile mode, centroided and analyzed using MATLAB (MathWorks). Missing values were filled by recursion in the raw data. Upon identification of consensus centroids across all samples, ions were putatively annotated by accurate mass and isotopic patterns. Based on the HMDB (version 4.0)^15^, a list of the expected ions found under these conditions was generated, including deprotonated, fluorinated and all major adducts. As this method does not employ chromatographic separation or in-depth MS2 characterization, it is not possible to distinguish between compounds with identical molecular formulas. The confidence of annotation reflects level 4 but is higher in practice in the case of intermediates of primary metabolism because they are the most abundant metabolites in cells^10,11^. Ion intensities were normalized by quantile normalization to compensate for slight variations in the sample amount. In this way, 1,183 peaks were annotated based on accurate mass using a 1-mDa tolerance (Supplementary Table 2). The annotated metabolites cover 18 chemical categories based on the HMDB^15^, including 341 lipids and lipid-like molecules, 218 organic acids and derivatives, 196 organoheterocyclic compounds, 118 phenylpropanoids and polyketides, 109 benzenoids, 104 organic oxygen compounds and 97 additional metabolites belonging to another 12 categories (Supplementary Table 2). Finally, we estimated the effect of sample plate batch on metabolite level and detected no batch effects.

To investigate potential factors that may influence the human plasma metabolome, we correlated the first 100 principal components of the 1,183 metabolites (accounting for 73% of the total metabolome variance) with age, sex, BMI, smoking, 78 dietary habits, 39 diseases and the use of 44 medications (Supplementary Table 22). As we were interested in the impact of diet, genetics and the gut microbiome on metabolites, we decided to correct for age, sex, smoking and oral contraceptive use, based on the correlation results. To adjust for confounding factors, we first log-transformed the metabolite abundances, then applied a linear regression model that included all of the confounding factors as covariates, taking the residuals for the subsequent analysis.

#### Stool microbiome

Fecal samples were collected by participants at home and placed in the freezer (−20 °C) within 15 min of production. Subsequently, a nurse visited the participant to pick up the fecal samples on dry ice and transfer them to the laboratory. Aliquots were then made and stored at −80 °C until further processing (fecal samples of the GoNL cohort were stored in RNAlater). The same protocol for fecal DNA isolation and metagenomics sequencing was used in all four cohorts. Fecal DNA isolation was performed using the AllPrep DNA/RNA Mini Kit (80204; Qiagen). After DNA extraction, fecal DNA was sent to the Broad Institute of MIT and Harvard, where library preparation and whole-genome shotgun sequencing were performed on an Illumina HiSeq platform. From the raw metagenomics sequencing data, low-quality reads were discarded by the sequencing facility and reads belonging to the human genome were removed by mapping the data to the human reference genome (version NCBI37) using KneadData (version 0.4.6.1) and Bowtie 2 (version 2.1.0)^57,58^.

Microbial taxonomic profiles were generated using MetaPhlAn2 (version 2.7.2)^59^. Microbial general pathways were determined using HUMAnN2 (ref. ^60^), which maps DNA/RNA reads to a customized database of functionally annotated pan-genomes. HUMAnN2 reported the abundances of gene families from the UniProt Reference Clusters^61^ (UniRef90), which were further mapped to microbial pathways from the MetaCyc metabolic pathway database^62,63^. In total, we detected 156 species and 343 pathways that were present in at least 10% of samples, retaining 98% of the original species composition and 100% of the original functional composition. The relative abundances of both species and pathway datasets were centered-log-ratio transformed, followed by inverse-rank transformation, before subsequent analysis^64^.

We applied the SGV-Finder pipeline^47^ to classify SVs that were either completely absent from the microbial genome of some samples (dSVs) or whose coverage was highly variable across samples (vSVs). Before SV classification, we applied an iterative coverage-based read assignment algorithm that resolves ambiguous read assignments to regions that are similar between different bacteria, using information on bacterial relative abundances in the microbiome, their genomic sequencing coverage and sequencing and alignment qualities^47^. In total, we classified 4,262 dSVs and 1,782 vSVs from 41 microbial species that were present in at least 10% of samples. The vSV data were inverse-rank transformed for subsequent analysis.

Metabolite-specific pathways were generated using the gutSMASH algorithm^46^. In total, we generated 3,075 microbial strain-level metabolite-specific pathways that were present in at least 10% of samples. The abundance of these pathways was recorded as reads per kilobase of transcript per million reads mapped, and inverse-rank transformation was applied before subsequent analysis.

#### Genotype data

Microarray genotype data for the LLD cohort were generated using the CytoSNP-12 Beadchip and Immunochip assays, as previously described^65^. Quality control checks on the LLD cohort were performed using the Haplotype Reference Consortium (version 1.0) preparation checking tool (version 4.2.3). We then uploaded the resulting VCF files to the Michigan Imputation Server^66^. Phasing and imputation were performed using the option SHAPEIT for phasing, population EUR and the mode Quality Control and Imputation. For all steps, we used version R1 as a reference^67^. We further excluded SNPs that had an imputation quality *r*^2^ < 0.5, failed the Hardy–Weinberg equilibrium test (*P* < 1 × 10^−6^), had a call rate of <95% or had a minor allele frequency of <5%. In total, we obtained genotype data for 5.3 million SNPs (genome build hg19) for 927 individuals after removing relatives and outliers. The genotypes of the GoNL samples were obtained by whole-genome sequencing.

### Statistical analysis

#### Distance matrix-based variance estimation

We applied feature selection based on the permutational multivariate analysis of variance using distance matrices (PERMANOVA) procedure to estimate the contributions of different factors to inter-individual variations of the whole plasma metabolome. Phenotypic, genetic and microbial contributions were estimated based on the 927 participants for whom plasma metabolites, a stool microbiome, phenotypic data and genotype were available. First, we used each raw phenotypic and microbial feature to estimate inter-individual metabolic variations using the adonis function from vegan (version 2.5.5) with 1,000 times permutation. Only phenotypic and microbial features that could estimate inter-individual metabolic variations at a permutational FDR of <0.05 were kept. For genetic variants, we used SNPs with significant mQTLs. To deal with the colinearity of selected features, we applied hierarchical clustering analysis based on a feature inter-correlation distance matrix (1 − *r*^2^). Features were assigned to different clusters based on 70% dissimilarity and the central feature in each cluster was selected as representative. All representative features were further included in PERMANOVA to estimate the combined contribution to inter-individual metabolome variation.

#### Associations with dietary habits

To assess associations between diet and metabolites, continuous dietary habits were inverse-rank transformed and corrected for age, sex and smoking. Spearman correlation was applied to assess the correlation between 78 dietary habits and 1,183 metabolites (residuals after regressing out age, sex, smoking and oral contraceptive use; Supplementary Table 2). The FDR was calculated using the Benjamini–Hochberg procedure^68^.

#### QTL mapping

This analysis involved the 927 participants for whom there were genotype, plasma metabolome and phenotypic data. After adjusting for the first two genetic principal components, age, sex, BMI, smoking, 78 dietary habits, 40 diseases and 44 medications, as described above, a Java-based pipeline (version 1.4nZ)^69^ was applied for QTL mapping by calculating the Spearman correlation between SNP dosage and metabolite residuals after regressing out the above covariates. We considered metabolite associations with *P* < 4.2 × 10^−11^ as significant—a threshold corresponding to a genome-wide significance cut-off of 5.0 × 10^−8^ divided by 1,183 tests. All independent mQTLs (clumping variants with linkage disequilibrium *r*^2^ < 0.05 and a 500-kb window^25^) above this threshold were reported. We also tested the impact of physical activities on QTL effect by re-running the QTL analysis in 855 Lifelines participants for whom we had SQUASH (the short questionnaire to assess health-enhancing physical activity) physical activity scores^24^.

#### Microbiome-wide associations

We previously reported that several medications can alter the gut microbiome significantly, including proton pump inhibitors, antibiotics and laxatives^70,71^. We therefore adjusted all microbial datasets for these confounding factors, together with age, sex and smoking. For microbial changes 4 years apart, we regressed out age and sex. Next, Spearman correlation was applied to check the associations between metabolites and microbial features, and *P* values were adjusted using the Benjamini–Hochberg procedure.

#### Interaction analysis

For metabolites associated with at least two types of factor (from genetics, the microbiome and diet), we further performed interaction analysis by assessing pairwise interactions between the two factors using a linear model (*y* = *a* + *b* + *a* × *b*). *P* values were adjusted using the Benjamini–Hochberg method.

#### Estimating the variance of individual metabolites

To estimate the variance of each metabolite that was contributed by dietary, genetic and microbial features, we applied machine learning-based lasso regression from the glmnet package (version 2.0.16). While ensemble machine learning methods have previously been shown to outperform the predictive capabilities of linear methods such as lasso^3^, lasso’s interpretability and capacity to integrate highly correlated data layers (microbiome taxa and dietary habits) made it an attractive methodology for our analysis. We believe that, while the overall variance explained might be an underestimation of the predictive power of the available data layers, the relative variability explained by each data layer should be representative of the dominant factor that explains most variance in each metabolite.

All of the dietary, microbial (general species and pathway relative abundance) and genetic features that were significantly associated with a specific metabolite at FDR < 0.05 were involved in the model. These features were further selected using lasso with a lambda that gave a minimum mean error from a tenfold cross-validation in order to control for overfitting and to provide a conservative estimate of model performance. Finally, features selected by lasso were included in the linear model to estimate the variance contributed by different factors, and the adjusted *r*^2^ and *F*-test *P* value were recorded. The FDR was calculated based on the Benjamini–Hochberg procedure. We also applied Elastic Net from the glmnet package (version 2.0.16) to estimate the variance of each metabolite contributed by different factors and compared the results with the above method.

#### Principal component analysis

The levels of all plasma metabolites were included in the principal component analysis. We applied the vegdist() function from the R package (version 2.5.5) vegan to calculate the Euclidean dissimilarity matrix based on the metabolite levels. Subsequently, classical metric multidimensional scaling was carried out based on the Euclidean distance matrix to obtain different principal coordinates.

#### Temporal consistency of individual metabolites

We used the Spearman correlation to check how consistent the levels of individual metabolites were between the baseline and 4-year follow-up. Metabolites with larger correlation coefficients were assumed to be more stable.

#### Lifelines diet quality score prediction

We first checked the Lifelines Diet Score^8^ associations with each metabolite in 1,054 LLD samples and selected the significant metabolite features (*P* < 0.05) for lasso regression, as described above. Metabolites selected by lasso were used to build the linear model, and 230 LLD_2_ samples with both diet score and plasma metabolome information available were used as validation. Adjusted *r*^2^ and the *P* value from the *F*-test were reported to reflect the performance of the prediction model. We also carried out these analyses for four other dietary scores: the alternate Mediterranean Diet Score^20^, HEI^21^, Protein Score^22^ and Modified Mediterranean Diet Score^23^.

#### Tissue-specific gene expression analysis

Summary statistics of independent mQTLs were used for tissue-specific gene expression analysis with FUMA^25^.

#### Bi-directional MR analysis

To evaluate whether the microbiome can causally contribute to plasma metabolites, we applied bi-directional MR analyses. A microbiome GWAS was performed using the same approach we applied for the metabolite GWAS, after correcting for age, sex, smoking and the use of proton pump inhibitors, antibiotics and laxatives. We focused on the 37 microbial features associated with at least three independent genetic variants at *P* < 1 × 10^−5^ in the baseline that could also be found in the follow-up samples with the same direction of association (*P* < 0.05) and on the 45 metabolites with significant associations with the microbiome (FDR_LLD1_ < 0.05 and *P*_LLD1 follow-up_ < 0.05). The relaxed significance threshold for choosing microbiome-associated SNPs as genetic instruments was used to increase the number of SNPs available for analyses, as described previously^9^.

MR analysis was done using the TwoSampleMR (version 0.5.5) R package. While this package was developed for two-sample MR analysis, a recent paper showed that it is possible to use most two-sample MR methods in the one-sample setting, including IVW^35^ and weighted median^72^. Therefore, MR estimates were calculated using Wald ratios and these Wald ratios were meta-analyzed using the IVW method^35^. In addition, we report MR estimates calculated using the weighted median test. We kept only the results based on three or more SNPs. To ensure the validity of the results, several sensitivity analyses were performed. We excluded MR estimates potentially driven by horizontal pleiotropy (removing results with MR-Egger^36^ intercept *P* < 0.05) and heterogeneity (removing results with Cohran’s *Q* test^35^ *P* < 0.05). In addition, we carried out leave-one-out analysis^73^ to check whether the MR estimates were possibly driven by a single SNP (removing the estimates where all but one leave-one-out configuration had *P* < 0.05). Multiple testing correction was performed using the Benjamini–Hochberg approach based on IVW *P* values. To avoid complex causality relationships, we excluded the results that showed a nominally significant MR estimate in the other direction (*P* < 0.05). For this analysis, metabolite-associated SNPs at a *P* value cut-off of 1 × 10^−5^ in the baseline group and *P* < 0.1 in the follow-up group were used as genetic instruments in IVW-based MR.

#### Bi-directional mediation analysis

For microbial features associated with both metabolites (FDR_LLD1_ < 0.05 and *P*_LLD1 follow-up_ < 0.05) and dietary habits (FDR < 0.05), we first checked whether the dietary habits were associated with the metabolite using Spearman correlation (FDR < 0.05). Next, we carried out bi-directional mediation analysis with interactions (*y* = *x* + *m* + *x* × *m*, where *y* is the outcome, *x* is the variable and *m* represents the mediator) between mediator and outcome taken into account using the mediate function from mediation (version 4.5.0) to infer the mediation effect of metabolites and microbiome for dietary impacts^74^. The FDR was calculated based on the Benjamini–Hochberg procedure.

### Reporting summary

Further information on research design is available in the Nature Research Reporting Summary linked to this article.

## Online content

Any methods, additional references, Nature Research reporting summaries, source data, extended data, supplementary information, acknowledgements, peer review information; details of author contributions and competing interests; and statements of data and code availability are available at 10.1038/s41591-022-02014-8.

## Supplementary information

Supplementary Information Supplementary Figs. 1–5.

Reporting Summary

Supplementary Tables Supplementary Tables 1–22.

## Data Availability

All processed microbiome abundance data and full summary statistics of mQTLs are freely available via the MOLGENIS cloud (https://geneticsresearch.molgeniscloud.org/menu/main/home), with an interactive browser of the top 100,000 mQTLs. The annotation of metabolites is based on the HMDB (https://hmdb.ca; version 5). The raw metagenomics sequence, metabolomics and basic phenotype data (age, sex and BMI) are deposited in the European Genome-phenome Archive (EGA) database with the study ID EGAS00001001704, which includes dataset IDs EGAD00001001991 (raw metagenomics sequencing) and EGAD00001006953 (raw metabolomics data). However, the use of Lifelines data and materials must comply with the informed consent signed by Lifelines participants specifying that their collected data will not be used for commercial purposes. There is a minimal access procedure for access to the EGA dataset that includes the provision of a contact address and completion of an online data access form (https://goo.gl/forms/TWHlrmbXaXNqWnnl2), which is very simple and is only intended to ensure that the data are being requested for research/scientific purposes only. Submitted data access forms will be evaluated by the data manager and Lifelines. For requests from verified academic parties, access will be granted within 2 weeks. There are no restrictions on downstream data re-use or authorship requirements. For requests from commercial parties, Lifelines will perform a pre-data protection impact assessment (pre-DIPA) to assess the risks of the proposed processing of personal data (for example, purpose, storage, access, archiving and so on) with respect to the General Data Protection Regulation (GDPR) subject rights. Based on the outcome of the pre-DPIA, Lifelines will decide whether sharing data with the commercial entity is allowed and/or whether additional measures have to be taken. Genotype and metadata, including disease, medication and other clinical and lifestyle information, are however privacy sensitive. To ensure adherence to participant’s privacy and informed consent, the rights of participants as described in the GDPR (EU privacy laws) and Lifelines biobank regulations, the complete genotype and phenotype data cannot be provided as open access and are only available from Lifelines under controlled access in a secure Lifelines Workspace or High-Performance Cluster (HPC) environment. As Lifelines is a non-profit organization dependent on (governmental) subsidies, a fee is required to cover the costs of controlled data access and supporting infrastructure. In brief, the step-by-step data access procedure is as follows: (1) data are requested by filling in the application form to request ‘Available Lifelines-data’ at https://www.lifelines.nl/researcher/how-to-apply/apply-here; (2) Lifelines will evaluate project proposals to ensure compliance with the Lifelines data access policy, the informed consent of Lifelines participants and the GDPR, and that the data are being requested for non-commercial research; (3) upon approval, Lifelines will send Data and Material Transfer Agreement contracts to the applicants; and (4) after the required contracts are signed, Lifelines will provide access to data via the Workspace or HPC and link the raw and processed Lifelines sequencing data to the Lifelines phenotypes. Lifelines strives to accomplish steps 2–4 at 2 weeks per step, assuming that no extra actions by the applicant or Lifelines are required. The fee for data access on the HPC is €3,500 for 1 year and the fee for the Lifelines Workspace environment is €4,500 for 1 year, or less for shorter periods of time. There are no restrictions on the downstream re-use of aggregated, non-identifiable results (as approved by Lifelines), nor are there authorship requirements, but Lifelines does request that it is acknowledged in publications using these data. The data access policy, data access fees and an example Data and Material Transfer Agreement (which includes details on how to acknowledge the use of Lifelines data in publications) are described in detail at https://www.lifelines.nl/researcher/how-to-apply. Note that data access for replication can be arranged via Lifelines. Lifelines will not charge an access fee for controlled access to the full dataset used in the manuscript (including phenotype and sequencing data) for a period of 3 months for the specific purpose of replication of the results presented in the current manuscript. Researchers interested in such a replication study can contact Lifelines at research@lifelines.nl. Further information can be obtained from Lifelines at https://www.lifelines.nl/researcher/how-to-apply/information-request or by contacting Lifelines at research@lifelines.nl.

## Code availability

Analysis codes are available via https://github.com/GRONINGEN-MICROBIOME-CENTRE/Groningen-Microbiome/tree/master/Projects/LLDeep_plasma_GeneralMeta.
