# Supplementary material for: Influence of the microbiome, diet and genetics on inter-individual variation in the human plasma metabolome
Source: Nat Med. 2022 Oct 10;28(11):2333–43. doi: 10.1038/s41591-022-02014-8 (PMC9671809; doi:10.1038/s41591-022-02014-8)

naturemedicine

Article

https://doi.org/10.1038/s41591-022-02014-8

Influence of the microbiome, diet and genetics on inter-individual variation in the human plasma metabolome

In the format provided by the authors and unedited

Supplementary information

Influence of the microbiome, diet and genetics on inter-individual variation in the human plasma metabolome

Table of Contents

Fig. S1 | Comparison of the proportion of variation explained by diet, microbes and genetics between the LASSO and ElasticNet feature selection methods 2

Fig. S2 | Baseline diet, genetics and gut microbiome estimate the inter-individual variation of the whole plasma metabolome in LLD follow-up 3

Fig. S3 | Comparison of mQTL effect sizes in different Lifelines cohorts 4

Fig. S4 | Comparison of mQTL effect sizes with and without adjustment for physical activity scores .. 5

Fig. S5 | Causal relationships between microbiomes and plasma metabolites with Weighted Median and Egger methods 6

Fig. S1 | Comparison of the proportion of variation explained by diet, microbes and genetics between the LASSO and ElasticNet feature selection methods

Each dot represents a metabolite. The x-axis indicates the explained variation at baseline and the y-axis indicates the explained variation at follow-up.

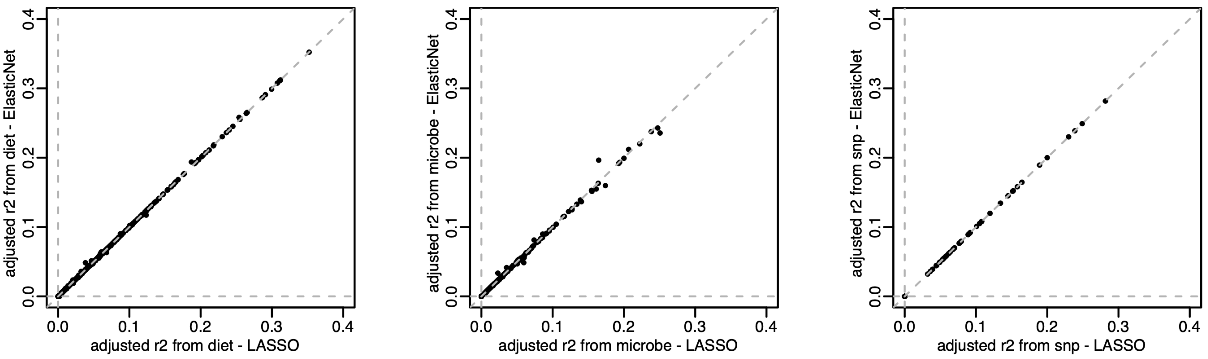

Fig. S2 | Baseline diet, genetics and gut microbiome estimate the inter-individual variation of the whole plasma metabolome in LLD follow-up.

The estimate of explained variation using the PERMANOVA method (two-sided)**.** The x-axis refers to explained variation in metabolic profile at the 2nd timepoint and the y-axis refers to models with different factors.

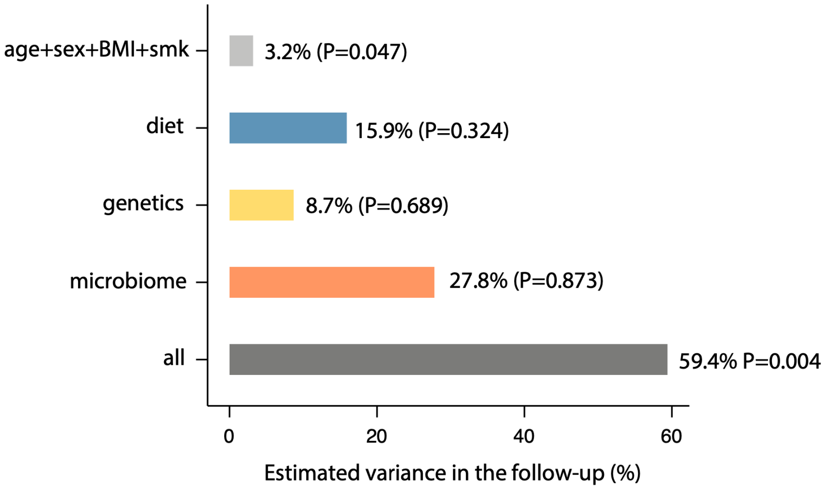

Fig. S3 | Comparison of mQTL effect sizes in different Lifelines cohorts

Dots represent independent mQTLs identified in the Lifelines-DEEP baseline samples. The x-axis indicates the effect size estimated in the LLD_1 cohort and the y-axis indicates the effect size estimated in the LLD_1-follow-up cohort (left), LLD_2 (middle) and GoNL samples (right). mQTLs significant at P < 0.05 level (Spearman, two-sided) in the corresponding cohort are shown in black. All others are in gray.

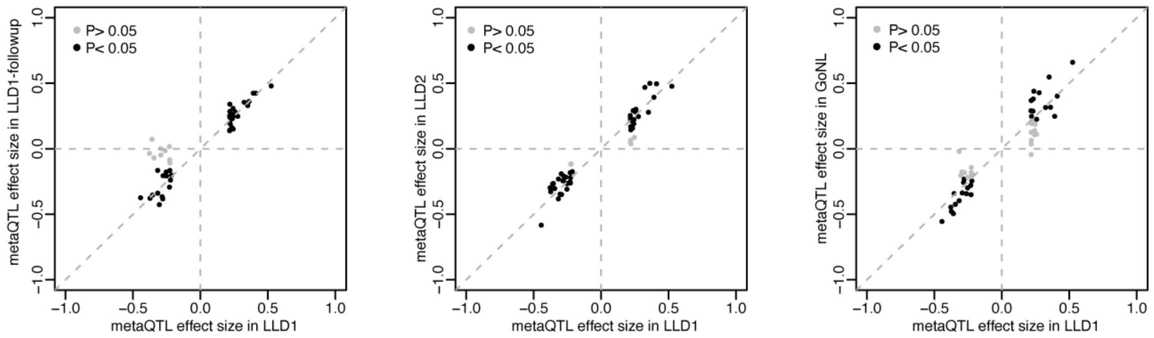

Fig. S4 | Comparison of mQTL effect sizes with and without adjustment for physical activity scores

Dots represent 3,008 study-wide significant mQTLs (from 927 samples) observed in QTL mapping without adjustment of physical activity scores. The x-axis indicates effect size estimated in the LLD_1 cohort (n=927 biologically independent samples) without adjustment for physical activity scores. The y-axis indicates effect size estimated in LLD_1 with adjustment for physical activity scores (n=855 biologically independent samples).

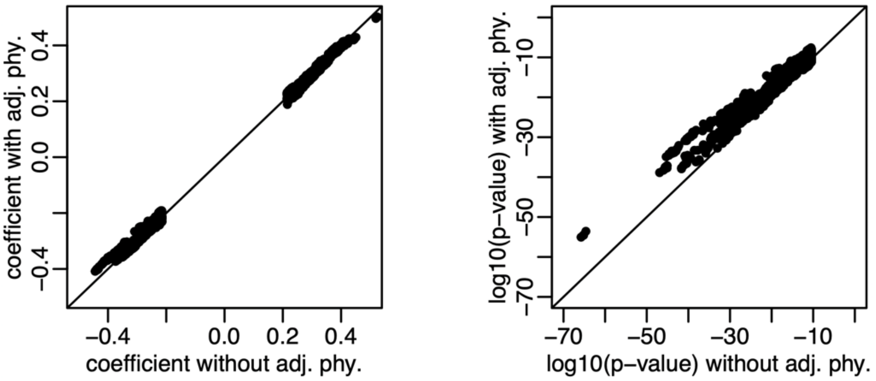

Fig. S5 | Causal relationships between microbiomes and plasma metabolites with Weighted Median and Egger methods

Forest plots show the MR effect size (center dot) and with 95% confidence intervals (CI) for the baseline (blue) and follow-up (green) datasets of the LLD_1 cohort, estimated by Weighted Median and Egger methods. The x-axis indicates effect size of MR. n=927 biological independent samples at baseline and n=311 biologically independent samples at follow-up.

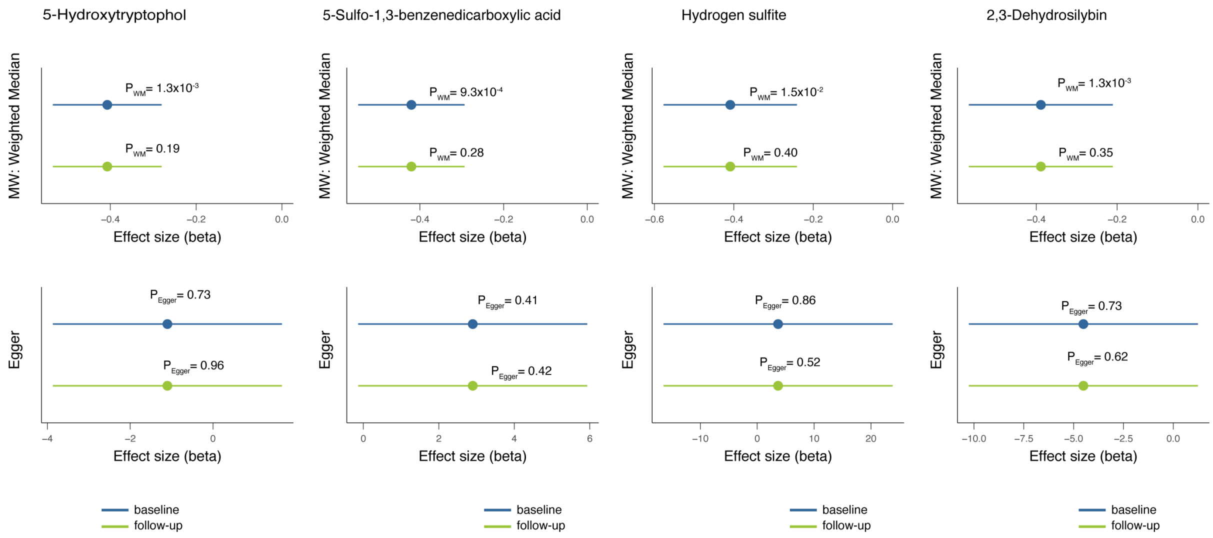

Supplement: Supplementary file 1 — Supplementary Figs. 1–5. [file 41591_2022_2014_MOESM1_ESM.pdf]
