# Supplementary material for: Influence of the microbiome, diet and genetics on inter-individual variation in the human plasma metabolome
Source: Nat Med. 2022 Oct 10;28(11):2333–43. doi: 10.1038/s41591-022-02014-8 (PMC9671809; doi:10.1038/s41591-022-02014-8)
Supplement: Supplementary file 2 — Reporting Summary [file 41591_2022_2014_MOESM2_ESM.pdf]

nature portfolio

Corresponding author(s): Jingyuan Fu

Last updated by author(s): Aug 11, 2022

Reporting Summary

Nature Portfolio wishes to improve the reproducibility of the work that we publish. This form provides structure for consistency and transparency in reporting. For further information on Nature Portfolio policies, see our Editorial Policies and the Editorial Policy Checklist.

Statistics

For all statistical analyses, confirm that the following items are present in the figure legend, table legend, main text, or Methods section.

n/a	Confirmed	
☐	☒	The exact sample size (*n*) for each experimental group/condition, given as a discrete number and unit of measurement
☐	☒	A statement on whether measurements were taken from distinct samples or whether the same sample was measured repeatedly
☐	☒	The statistical test(s) used AND whether they are one- or two-sided<br>*Only common tests should be described solely by name; describe more complex techniques in the Methods section.*
☐	☒	A description of all covariates tested
☐	☒	A description of any assumptions or corrections, such as tests of normality and adjustment for multiple comparisons
☐	☒	A full description of the statistical parameters including central tendency (e.g. means) or other basic estimates (e.g. regression coefficient) AND variation (e.g. standard deviation) or associated estimates of uncertainty (e.g. confidence intervals)
☐	☒	For null hypothesis testing, the test statistic (e.g. *F*, *t*, *r*) with confidence intervals, effect sizes, degrees of freedom and *P* value noted<br>*Give P values as exact values whenever suitable.*
☒	☐	For Bayesian analysis, information on the choice of priors and Markov chain Monte Carlo settings
☒	☐	For hierarchical and complex designs, identification of the appropriate level for tests and full reporting of outcomes
☐	☒	Estimates of effect sizes (e.g. Cohen's *d*, Pearson's *r*), indicating how they were calculated

Our web collection on statistics for biologists contains articles on many of the points above.

Software and code

Policy information about availability of computer code

Data collection	No specific software was used for data collection.
Data analysis	All data analyses were conducted using publicly available tools. For this study the following software was used: kneadData (v0.4.6.1), Bowtie2 (v2.1.0), MetaPhlAn2 (v2.7.2), HUMAnN2 (v0.10.0), gutSMASH*, SGV-Finder*, vegan (v2.5.5), glmnet (v2.0.16), TwoSampleMR (v0.5.5), mediation (v4.5.0). Code used for data analyses is publicly available at: [https://github.com/GRONINGEN-MICROBIOME-CENTRE/Groningen-Microbiome/tree/master/Projects/LLDeep_plasma_GeneralMeta]. * No version number available.

For manuscripts utilizing custom algorithms or software that are central to the research but not yet described in published literature, software must be made available to editors and reviewers. We strongly encourage code deposition in a community repository (e.g. GitHub). See the Nature Portfolio guidelines for submitting code & software for further information.

Data

Policy information about availability of data

All manuscripts must include a data availability statement. This statement should provide the following information, where applicable:

- Accession codes, unique identifiers, or web links for publicly available datasets
- A description of any restrictions on data availability
- For clinical datasets or third party data, please ensure that the statement adheres to our policy

All processed microbiome abundance data and full summary statistics of mQTLs are freely available via the MolGenis cloud (https://

geneticsresearch.molgeniscloud.org/menu/main/home), with an interactive browser of the top 100,000 mQTLs. The annotation of metabolites is based on the Human Metabolome Database (https://hmdb.ca, version 5). Tissue specific expression of genes is based on the Genotype-Tissue Expression (GTEx) database (https://gtexportal.org/home, version 8). The raw metagenomics sequence, metabolomics, and basic phenotype data (age, sex and BMI) are deposited at the EGA database with the study ID EGAS00001001704 (https://ega-archive.org/studies/EGAS00001001704), which include Dataset ID EGAD00001001991 for raw metagenomics sequencing (https://ega-archive.org/datasets/EGAD00001001991 and dataset ID EGAD00001006953 for raw metabolomics data (https://ega-archive.org/datasets/EGAD00001006953). However, the use of Lifelines data and materials must comply with the informed consent signed by Lifelines participants specifying that their collected data will not be used for commercial purposes. There is a minimal access procedure for the access of the EGA dataset that includes a contact address and a online data access form https://goo.gl/forms/TWHlrmbXaXNqWnnl2, which is very simple and is only intended to ensure that the data is being requested for research/scientific purposes only. Submitted data access forms will be evaluated by the data manager and Lifelines. For requests from verified academic parties, access will be granted within two weeks. There are no restrictions on downstream data re-use or authorship requirements. For requests from commercial parties, Lifelines will perform a pre-DPIA (Data Privacy Impact Assessment) to assess the risks of the proposed processing of personal data (e.g. purpose, storage, access, archiving, etc.) with respect to the GDPR subject rights. Based on the outcome of the pre-DPIA, Lifelines will decide whether sharing data with the commercial entity is allowed and/or whether additional measures have to be taken.
Genotype and metadata, including disease, medication and other clinical and lifestyle information, are however privacy sensitive. To ensure adherence to participant's privacy and informed consent, the rights of participants as described in the GDPR (EU privacy laws) and Lifelines biobank regulations, the complete genotype and phenotype data cannot be provided open-access and is only available from the Lifelines under controlled-access in a secure Lifelines Workspace or High Performance Cluster (HPC) environment. As Lifelines is a non-profit organization dependent on (governmental) subsidies, a fee is required to cover the costs of controlled data access and supporting infrastructure.
In brief, the step-by-step data access procedure is as follows: 1) Data is requested by filling the application form to request "Available Lifelines-data" at https://www.lifelines.nl/researcher/how-to-apply/apply-here; 2) Lifelines will evaluate project proposals to ensure compliance with the Lifelines data access policy, informed consent of Lifelines participants and the GDPR and that the data is being requested for non-commercial research; 3) Upon approval, Lifelines will send Data and Material Transfer Agreement (DMTA) contracts to the applicants; and 4) After the required contracts are signed, Lifelines will provide access to data via the Workspace or HPC and link the raw and processed DMP sequencing data to the Lifelines phenotypes. Lifelines strives to accomplish steps 2–4 at 2-weeks per step, assuming that no extra actions by the applicant or Lifelines are required.
The fee for data access on the HPC is €3,500 for one year and the fee for the Lifelines Workspace environment is €4,500 for one year, or less for shorter periods of time. There are no restrictions on downstream re-use of aggregated, non-identifiable results (as approved by Lifelines), nor are there authorship requirements, but Lifelines does request that it is acknowledged in publications using these data.
The data access policy, data access fees and an example DMTA (which includes details on how to acknowledge the use of Lifelines data in publications) are described in detail at https://www.lifelines.nl/researcher/how-to-apply. Note, data access for replication can be arranged via the Lifelines. Lifelines will not charge an access fee for controlled access to the full dataset used in the manuscript (including phenotype and sequencing data) for a period of 3 months, for the specific purpose of replication of the results presented in the current manuscript. Researchers interested in such a replication study can contact Lifelines at research@lifelines.nl. Further information can be obtained from Lifelines at https://www.lifelines.nl/researcher/how-to-apply/information-request or by contacting Lifelines at research@lifelines.nl.

Human research participants

Policy information about studies involving human research participants and Sex and Gender in Research.

Reporting on sex and gender	The study includes both sexes, which is balanced as much as possible and with the male to female ratio of 41.8 to 58.2. Sex was included as a covariant for association analyses, and no sex-specific analysis was performed.
Population characteristics	The study has included the population-based cohorts (LLD1, LLD2 and GoNL) from the Netherlands, n=1138, 58.20% female, the mean age (SD) of participants is 45.04 (SD 13.60) years and their mean BMI is 25.26 (SD 4.18).
Recruitment	All participants cohorts (LLD1, LLD2, GoNL) are part of the prospective, population-based LifeLines cohort. From April to August 2013, all participants registered at the LifeLines Research Site in Groningen were invited to participate in the study, a study with deep omics profiling in addition to the regular LifeLines programme. All participants were collected without any specific selection. Most participants were from the north of the Netherlands and thus the reported results could be region-specific.
Ethics oversight	All participants signed an informed consent form prior to sample collection. The Medical Ethical Commitee of the University Medical Center Groningen (UMCG) has approved the study with reference number M12.113965. The Medical Ethical Committee (in Dutch: Medisch Ethische Toetsingsingscommissie or METc) of UMCG evaluates protocols for scientific research involving human beings. Such evaluation was given a legal context when on 1 December 1999, the Law on Medical Scientific Research involving Human Beings (WMO) took effect. The METc is authorized to evaluate research that is conducted by the UMCG. For any questions, please contact: +31 50 – 361 4204

Note that full information on the approval of the study protocol must also be provided in the manuscript.

Field-specific reporting

Please select the one below that is the best fit for your research. If you are not sure, read the appropriate sections before making your selection.

☒ Life sciences ☐ Behavioural & social sciences ☐ Ecological, evolutionary & environmental sciences

For a reference copy of the document with all sections, see nature.com/documents/nr-reporting-summary-flat.pdf

Life sciences study design

All studies must disclose on these points even when the disclosure is negative.

Sample size	In total, this study includes 1,053 individuals of the Lifelines-DEEP (LLD1), 237 individuals from the LLD2 and 77 individuals from the GoNL cohorts, for whom we had collected extensive phenotypic datasets. Moreover, 311 individuals from the LLD1 cohort were followed up 4 years later. In order to ensure the analysis power, the study includes as much as subjects as possible. Thus no sample size calculation was performed. Detailed cohort description and sample size are shown in the Extended Data Fig 1.
Data exclusions	Only samples with missing metagenomics, metabolomics, genetics or dietary habits were excluded in analyses.
Replication	The LLD1 cohort served as a discovery cohort. Two independent replications were done using two independent cohorts (LLD2 and GoNL), respectively. One non-independent replication was done using the follow-up samples of the LLD1 cohort, which were profiled again 4 years later. Detailed replication scheme is shown in Extended Data Fig 1.
Randomization	This is human cohort-based analysis. The sample collection and sequencing were performed in a random order. No extra randomization was done for this study.
Blinding	This study is a human cohort based, observational study. Thus no blinding was performed.

Reporting for specific materials, systems and methods

We require information from authors about some types of materials, experimental systems and methods used in many studies. Here, indicate whether each material, system or method listed is relevant to your study. If you are not sure if a list item applies to your research, read the appropriate section before selecting a response.

Materials & experimental systems

n/a	Involved in the study	
☒	☐	Antibodies
☒	☐	Eukaryotic cell lines
☒	☐	Palaeontology and archaeology
☒	☐	Animals and other organisms
☒	☐	Clinical data
☒	☐	Dual use research of concern

Methods

n/a	Involved in the study	
☒	☐	ChIP-seq
☒	☐	Flow cytometry
☒	☐	MRI-based neuroimaging
